# Clarithromycin Attenuates Radiation-Induced Lung Injury in Mice

**DOI:** 10.1371/journal.pone.0131671

**Published:** 2015-06-26

**Authors:** Seung Jun Lee, Chin-ok Yi, Rok Won Heo, Dae Hyun Song, Yu Ji Cho, Yi Yeong Jeong, Ki Mun Kang, Gu Seob Roh, Jong Deog Lee

**Affiliations:** 1 Division of Pulmonary and Critical Care Medicine, Department of Internal Medicine, Gyeongsang National University Hospital, Gyeongsang National University School of Medicine, Jinju, Republic of Korea; 2 Department of Anatomy & Convergence Medical Science, Institute of Health Sciences, Gyeongsang National University School of Medicine, Jinju, Republic of Korea; 3 Department of Pathology, Gyeongsang National University Hospital, Gyeongsang National University School of Medicine, Jinju, Republic of Korea; 4 Department of Radiation Oncology, Gyeongsang National University Hospital, Gyeongsang National University School of Medicine, Jinju, Republic of Korea; Chinese Academy of Sciences, CHINA

## Abstract

Radiation-induced lung injury (RILI) is a common and unavoidable complication of thoracic radiotherapy. The current study was conducted to evaluate the ability of clarithromycin (CLA) to prevent radiation-induced pneumonitis, oxidative stress, and lung fibrosis in an animal model. C57BL/6J mice were assigned to control, irradiation only, irradiation plus CLA, and CLA only groups. Test mice received single thoracic exposures to radiation and/or oral CLA (100 mg/kg/day). Histopathologic findings and markers of inflammation, fibrosis, and oxidative stress were compared by group. On a microscopic level, CLA inhibited macrophage influx, alveolar fibrosis, parenchymal collapse, consolidation, and epithelial cell changes. The concentration of collagen in lung tissue was lower in irradiation plus CLA mice. Radiation-induced expression of tumor necrosis factor (TNF)-α, TNF receptor 1, acetylated nuclear factor kappa B, cyclooxygenase 2, vascular cell adhesion molecule 1, and matrix metallopeptidase 9 were also attenuated by CLA. Expression levels of nuclear factor erythroid 2-related factor 2 and heme oxygenase 1, transforming growth factor-β1, connective tissue growth factor, and type I collagen in radiation-treated lungs were also attenuated by CLA. These findings indicate that CLA ameliorates the deleterious effects of thoracic irradiation in mice by reducing pulmonary inflammation, oxidative damage, and fibrosis.

## Introduction

Radiation is an indispensable therapeutic modality for treating thoracic malignancies, including various cancers (e.g., lung, esophagus, head and neck, and breast), thymoma, malignant pleural mesothelioma, and lymphoma [1]. Unfortunately, radiation-induced cell death is not confined to tumors. Normal lung tissue is injured as well. To minimize collateral damage and maximize doses of radiation delivered to tumor cells, a variety of techniques have been developed. Advanced methods, such as three-dimensional (3D) radiotherapy and intensity-modulated radiotherapy (IMRT) have improved outcomes in patients with lung cancer [2,3]. More recently, proton therapy has prolonged survival without significant radiation pneumonitis and has reduced radiation doses to normal tissues in patients with non-small cell lung cancer [4]. Nevertheless, none of these techniques completely protects normal lung tissues from radiation effects.

Because toxic injury of normal tissues adjacent to the tumor cells is inevitable, and clinically significant radiation-induced lung injury (RILI) often occurs, physicians must be cognizant of risk factors and preventive strategies. An array of dosimetric and clinical predictors of RILI have been evaluated to this end. Mean lung dose, volume of lungs exposed to a specific dose, and normal tissue complication probability are commonly used dosimetric indices [5,6]. Clinical indices include pulmonary function, smoking status, age, performance status, combination chemotherapy, and tumor location [1,6,7].

Similarly, a number of pharmacologic agents intended to prevent RILI have been tested, and experimental agents are still under investigation. Previous studies have shown that amifostine, captopril, and pentoxifylline may have cytoprotective activity against radiation injury [6,8–10]. Antonadou et al. found that administration of amifostine significantly reduced RILI [8,11]. However, no significant reduction in radiation pneumonitis or esophagitis was achieved by Movsas et al. through amifostine use [12]; and radioprotective effects of other experimental agents have been anecdotal. Furthermore, none of these agents has been approved or is routinely used for prevention of RILI.

Clarithromycin (CLA) is a macrolide antibiotic prescribed for various respiratory infections, including pneumonia, chronic obstructive pulmonary disease, pharyngitis, and tonsilitis. Macrolides exert antibacterial activity by inhibiting bacterial protein synthesis. Although mounting evidence suggests that macrolides may profoundly impact the inflammatory milieu and immune responsiveness [13,14], mechanisms integral to their anti-inflammatory/immunomodulatory properties remain unclear. Various cytokines, chemokines, nuclear transcription factors, and reactive oxygen species apparently are involved [13]. Macrolides heighten mucocilliary clearance, diminish mucus secretion, and reduce bronchial hyper-responsiveness [14]. Macrolides also enhance or reduce levels of various cytokines and chemokines to regulate the immune response [14].

It was our contention that CLA might prevent RILI by dampening radiation-induced acute and chronic inflammation and ensuing immune response-related fibrosis of the lung. Consequently, the effects of CLA on radiation-induced pulmonary inflammation, fibrosis, and oxidative damage were investigated in a mouse animal model and are reported herein.

## Materials and Methods

### Ethics Statement

The experiments were approved (GLA-110603-M0035) by the Gyeongsang National University Institution Animal Care & Use Committee.

### Experimental animals

Female C57BL/6J mice (8-week-old; KOATEC, Pyeongtaek, Korea) were used for all studies and maintained in the animal facility at Gyeongsang National University School of Medicine, and our Board of Research approved the study protocol. The experiments were performed in accordance with the National Institutes of Health Guidelines on the Use of Laboratory Animals. Mice were housed five per cage and were provided standard rodent chow and water *ad libitum*. A total of 27 animals were randomly divided into four groups: control (CTL, n = 7), irradiation only (RT, n = 9), irradiation plus CLA (RT + CLA, n = 9), and CLA only (CLA, n = 2).

### Irradiation and clarithromycin treatment

We combined 2% xylazine hydroxychloride (3 mg/kg, Rompun; Bayer Korea Ltd., Seoul, Korea) and tiletamine-zolazepam (2 mg/kg, Zoletil 50; Virbac Laboratories, Carros, France) for intramuscular injection prior to irradiation. Mice were positioned to completely irradiate thorax cages. A 3-cm block of Lucite was placed above the chest to provide adequate buildup and facilitate even distribution of radiation. The mice were then subjected to single 18-Gy exposures using a 4MV linear accelerator (21EX 3153 VARIAN, Palo Alto, CA, USA). Oral CLA (100 mg/kg/day; Sandoz Industrial Products S.A., Barcelona, Spain) was administered five times weekly, starting 1 week before irradiation and continuing until the day of sacrifice. Two groups of mice (RT + CLA and CLA) received oral CLA for a total of 17 weeks.

### Preparation of lung tissues

The test mice were sacrificed at Week 16 post-irradiation under general anesthesia (Zoletil 5 mg/kg; Virbac Laboratories), and midline thoracotomies were performed. Right lungs were removed and stored at -80°C for later study. Left lungs were extracted for histologic examinations.

### Histopathology and fibrosis score

Left lung tissues were fixed with 4% paraformaldehyde in 0.1 M phosphate-buffered saline (PBS), embedded in paraffin, and sectioned (5-*μ*m thickness) for routine hematoxylin and eosin (H&E) and Sirius red staining, the latter highlighting histopathologic changes such as fibrosis. Degree of lung fibrosis was scored on a scale from 0 to 5, which was modified from a previous report [15]. Grade 0 represents normal lung. Grade 1 to 5 means minimal fibrosis with thickening of alveolar wall to totally obliterative fibrosis. Histologic derangements were also scored (1, minimal; 2, moderate; 3, extensive), as previously described [16]. Macrophage influx and parenchymal cell changes including collapse and consolidation, alveolar epithelial cell changes, and bronchial epithelial cell alterations were graded. Regarding macrophage influx, 1 denotes mild influx; 2 denotes moderate influx; 3 denotes extensive influx. Regarding parenchymal changes, 1 denotes less than 10% of lung parenchymal changes; 2 denotes 10–50% of lung parenchymal changes; 3 denotes more than 50% of lung parenchymal changes. All scores were assigned by one expert pathologist and one clinician blinded to the study design and outcomes. To quantify the Sirius red stained collagen in an image of lung sections (n = 2–6 mice per group), we used the Image J (NIH image program, version 1.44) software. The percentage of fibrosis was assessed by determining the total tissue areas occupied by pneumocytes and collagen and excluding empty spaces, as wells as excluding annotations of perivascular fibrosis and staining artefacts.

### Sircol collagen assay

The Sircol assay kit (Biocolor Ltd., Belfast, Northern Ireland, UK) was utilized to assess collagen content of lung tissue. Briefly, the specimens previously frozen in liquid nitrogen and stored at -80°C were thawed, and assays were performed as instructed by the manufacturer. Collagen concentrations were determined from a standard curve.

### Immunohistochemical analysis

Sections of lung tissue were sequentially deparaffinized, treated with 1% H_2_O_2_ (10 min), and thoroughly rinsed with PBS. Non-specific binding of immunoglobulin G (IgG) was eliminated by applying 2% normal goat serum in PBS (60 min, room temperature). Sections were then incubated with various primary antibodies, including rabbit anti-cyclooxygenase-2 (COX-2, 1:100; Cayman Chemical Co., Ann Arbor, MI, USA) and rabbit anti-heme oxygenase-1 (HO-1, 1:100; Enzo Clinical Labs Inc., Farmingdale, NY, USA). The sections were washed with PBS, incubated at room temperature for 60 min with biotin-conjugated secondary IgG (1:200; Vector Laboratories Inc., Burlingame, CA, USA), diluted via 2% normal blocking serum, and incubated again (60 min, room temperature) with avidin-biotin-peroxidase complex (Vectastain Elite ABC kit; Vector Laboratories). Finally, they were washed with PBS and incubated (3 min) in diaminobenzidine tetrahydrochloride (Sigmafast DAB; Sigma-Aldrich Co, St. Louis, MO, USA) containing 0.05% hydrogen peroxidase to develop color. All sections were examined via light microscopy (Model BX51; Olympus, Tokyo, Japan). Digital images were captured and analyzed. To automate immunohistochemistry scoring for HO-1 and COX-2 from lung sections (n = 2–6 mice per group), we used Image J software, as previously described [17]. Four random views in each group were selected to detect the intensity values. Based on the mean range of intensity values, each image was normalized by adjusting each color range of the image using Image J. Intensity measurements are represented as the percentage of mean number of pixels vs. the corresponding value at which the pixel of the respective intensity was present.

### Western blot analysis

Total right lung extractswere chopped in ice-cold lysis buffer (10 mM HEPES-KOH [pH7.9], 1.5 mM MgCl_2_, 10 mM KCl, protease inhibitors), homogenized, and centrifuged for 1 min at 12,000 rpm. Protein concentrations of lysates were determined using bovine serum albumin (BSA) standards and bicinchoninic acid (BCA) kits (Pierce Biotechnology Inc./Thermo Fisher Scientific, Rockford, IL, USA). Equal amounts (30 g) of protein were separated by sodium dodecyl sulfate-polyacrylamide gel electrophoresis and transferred to nitrocellulose membranes. The membranes were washed in Tris-buffered saline containing 0.5% Tween-20 (TBST) and incubated with the following TBST-diluted primary antibodies: rabbit anti-cleaved caspase-3 (Cell signaling, Beverly, MA, USA), goat anti-tumor necrosis factor (TNF)-α, mouse anti-TNF receptor 1 (TNFR1), and rabbit anti-TNFR2 (Santa Cruz Biotechnology Inc., Santa Cruz, CA, USA); rabbit anti-acetylated nuclear factor (NF)-κB p65 (Abcam, Cambridge, UK); rabbit anti-COX-2 (Cayman Chemical); rabbit anti- nuclear factor erythroid 2-related factor 2 (Nrf2; Santa Cruz Biotechnology); rabbit anti-HO-1 (Enzo Clinical Labs); rabbit anti-matrix metallopeptidase 9 (MMP-9; EMD Millipore, Temecula, CA, USA); rabbit anti-vascular cell adhesion molecule-1 (VCAM-1; Santa Cruz Biotechnology); rabbit anti-transforming growth factor β1 (TGF-β1; Santa Cruz Biotechnology); rabbit anti-connective tissue growth factor (CTGF; Abcam); rabbit anti-collagen type I (Calbiochem, La Jolla, CA, USA); mouse anti-β-actin (Sigma-Aldrich). All samples were then incubated with their corresponding secondary antibodies. Target proteins were visualized using an enhanced chemiluminescence western blot analysis system (Amersham Pharmacia Biotech Inc./GE Healthcare, Piscataway, NJ, USA). To determine relative amounts of protein, β-actin level was used as an internal control.

### Statistical analysis

Data are presented as mean ± standard error of mean (SEM). One-way analysis of variance (ANOVA) was applied to compare continuous variables between groups, followed by the Student-Newman-Keul test. Standard software (SPSS v18.0 for Windows; SPSS Inc., Chicago, IL, USA) was utilized for all analyses, setting statistical significance at *p*<0.05.

## Results

### Effects of CLA administration on body weight and mortality in irradiated mice

We hypothesized that CLA exerts an anti-inflammatory effect in irradiated mouse lung. To assess the effects of CLA administration on radiation-induced inflammation, oral CLA (100 mg/kg/day) treatment was initiated in mice 1 week prior to single radiation exposure and continued for a total of 17 weeks. At week 16 post-irradiation, mean body weight of RT mice (21.88 ± 0.95) was significantly lower than mean weight of CTL mice (27.75 ± 1.75, *p* < 0.05). Mean weight of mice in the RT + CLA group was 23.67 ± 0.88. Three of nine RT mice (33.3%) expired between weeks 15 and 16 post-irradiation. However, all mice of RT + CLA and CLA groups survived until the day of sacrifice.

### Effects of CLA administration on histopathologic features and apoptosis of RILI

**Fig 1:**
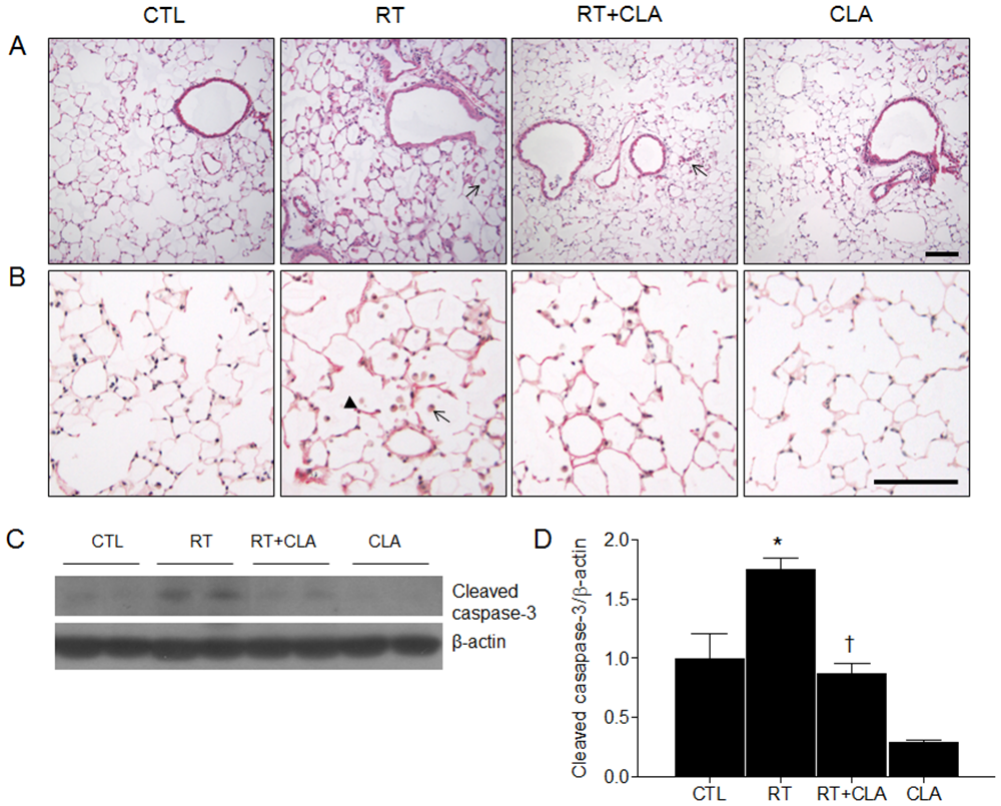
Effects of clarithromycin on radiation-induced macrophage influx, alveolar septal changes, and apoptosis in lungs of mice. (A) Representative photomicrographs of H&E-stained lung sections from control (CTL), radiation only (RT), radiation + clarithromycin (RT+CLA), and clarithromycin only (CLA) animal groups (macrophage at arrow). (B) Representative photomicrographs of sirius red-stained lung sections from each group. Thin arrow indicates macrophage and bold arrow indicates thickened, fibrotic alveolar septum). Scale bar = 100μm. (C) Cleaved caspase-3 expression in lungs of control (CTL), radiation only (RT), radiation + clarithromycin (RT+CLA), and clarithromycin only (CLA) animal groups. (D) Cleaved caspase-3 expression in lungs of respective groups. Densitometry values were normalized to β-actin and data are presented as mean ± SEM (n = 2–6 mice per group). *p<0.05 vs CTL mice; †*p*<0.05 vs RT mice.

Histopathologic changes were assesed in H&E- and Sirius red-stained lung sections (Fig 1A and 1B). Automated fibrosis scoring from the sirius red stained lung sections showed that the percentage of fibrosis in RT mice (185.51 ± 15.51) was increased compared with CTL mice (100.00 ± 12.10), whereas it was significantly inhibited by CLA administration (141.87 ± 8.11, *p* < 0.05). Compared with CTL mice, the influx of macrophages and interstitial edema in histologic sections were much greater in RT mice. However, both manifestations were inhibited by CLA administration. Likewise, scoring of macrophage influx, parenchymal collapse, consolidation, and alveolar epithelial cell changes were significantly higher in RT mice (vs CTL mice) and lower in RT + CLA mice (vs RT mice). Relative to CTL mice, fibrosis scores of RT mice were significantly higher (4.00 ± 0.24 vs 0.71 ± 0.18, *p* < 0.001); fibrosis scores of RT + CLA mice were significantly lower than those of RT mice (1.44 ± 0.53 vs 4.00 ± 0.24, *p* < 0.001) (Table 1). Radiation causes alveolar inflammatory cell apoptosis in lung injury [18]. To evaluate the effect of CLA on apoptosis in RILI, we performed western blot analysis using antibody to cleaved caspase-3, active caspase-3 (Fig 1C). We found that the expression level of cleaved caspase-3 in RT mice lung tissue were elevated compared with corresponding levels in lungs of CTL mice. However, its expression in RT mice was significantly reduced by CLA administration (Fig 1D).

**Table 1:** Histopathologic and fibrosis scores by group.

	CTL(n = 7)	RT (n = 6)	RT+CLA (n = 9)	CLA (n = 2)	p-value
Macrophage influx	1.00 ± 0.00	2.17 ± 0.41	1.67 ± 0.50	1.58 ± 0.71	0.001
Parenchymal cell changes	1.00 ± 0.00	2.11 ± 0.20	1.33 ± 0.24	1.00 ± 0.00	0.002
Lung fibrosis	0.71 ± 0.18	4.00 ± 0.24	1.44 ± 0.53	0.00 ± 0.00	< 0.001

CTL, control; RT, radiation: CLA, clarithromycin

### Impact of CLA administration on inflammation in irradiated lungs

**Fig 2:**
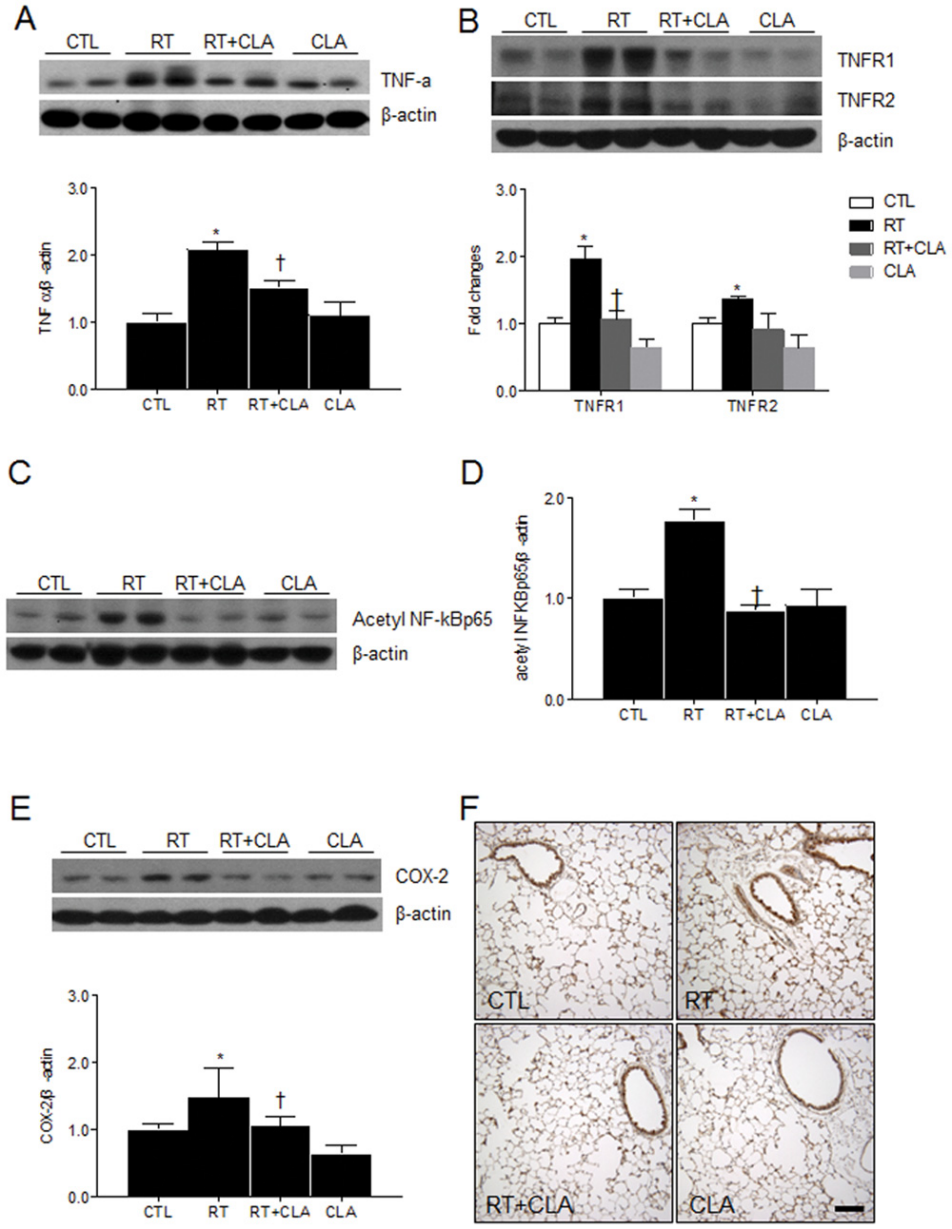
Effects of clarithromycin on inflammation in irradiated lungs of mice. (A) TNF-α expression in lungs of control (CTL), radiation only (RT), radiation + clarithromycin (RT + CLA), and clarithromycin only (CLA) animal groups. (B) TNFR1 and TNFR2 expression in lungs of respective groups. (C and D) Acetylated NF-κB p65 and (E) COX-2 expression in lungs of respective groups. Densitometry values were normalized to β-actin and data are presented as mean ± SEM (n = 2–6 mice per group). *p<0.05 vs CTL mice; †*p*<0.05 vs RT mice. (F) Immunostained COX-2 in lung tissue by group. Scale bar = 100 μm.

To investigate the effect of CAL on radiation-induced pro-inflammatory cytokine and its receptors, expression levels of TNF-α, TNFR1, and TNFR2 were measured. We found that the expression levels of TNF-α, TNFR1, and TNFR2 in RT mice lung tissue were elevated compared with corresponding levels in lungs of CTL mice. However, concomitant administration of CLA with thoracic irradiation significantly reduced expression levels of TNF-α, TNFR1, and TNFR2 (Fig 2A and 2B).

To evaluate whether inflammatory cytokine affect NF-κB-mediated pathway in irradiated lung, we confirmed acetylation of NF-κB and COX-2 expression using western blot analysis. Relative to CTL mice, CLA administration inhibited both the increased acetylation of NF-κB p65 (Fig 2C and 2D) and the elevated expression of COX-2 (Fig 2E) observed in the lung tissue of RT mice. CLA suppression of COX-2 expression was also verified immunohistochemically, contrasting with the elevated immunoreactivity of COX-2 in RT lung specimens (Fig 2F). In agreement with western blot analysis, we found that the percentage of mean intensity of COX-2 in RT mice was 267.84 ± 24.03. However, the percentage in RT+CLA mice (120.51 ± 39.86) was significantly reduced compared to RT mice (*p* < 0.05).

### Impact of CLA administration on VCAM-1 and MMP-9 expression levels in irradiated lungs

**Fig 3:**
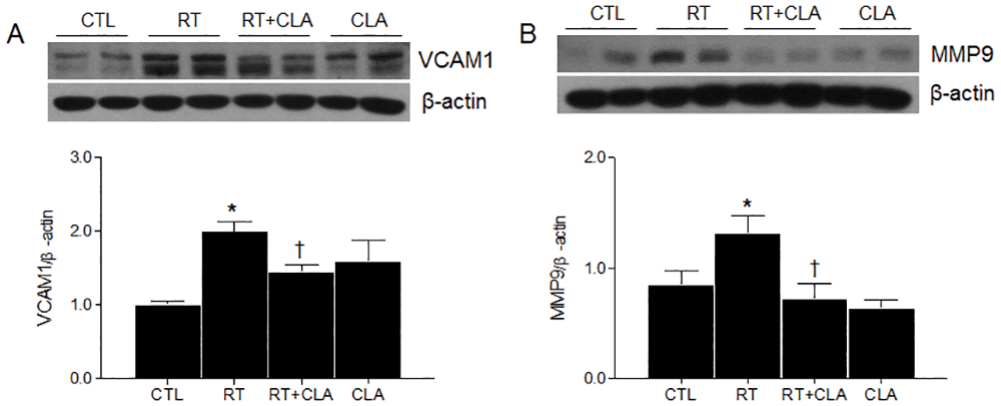
Effects of clarithromycin on VCAM-1 and MMP-9 expression levels in irradiated lungs of mice. (A) VCAM-1 expression in lungs of control (CTL), radiation only (RT), radiation + clarithromycin (RT+CLA), and clarithromycin only (CLA) animal groups. (B) MMP-9 expression in lungs of respective groups. Densitometry values were normalized to β-actin and data are presented as mean ± SEM (n = 2–6 mice per group). *p<0.05 vs CTL mice; †*p*<0.05 vs RT mice.

In addition to COX-2 expression, target proteins of acetylated NF-κB include VCAM-1 and MMP-9. Western blot analyses showed increased expression levels of VCAM-1 and MMP-9 in lungs of RT mice. However, such elevations were attenuated by CLA administration (Fig 3).

### Impact of CLA administration on Nrf2 and HO-1 expression levels in irradiated lungs

**Fig 4:**
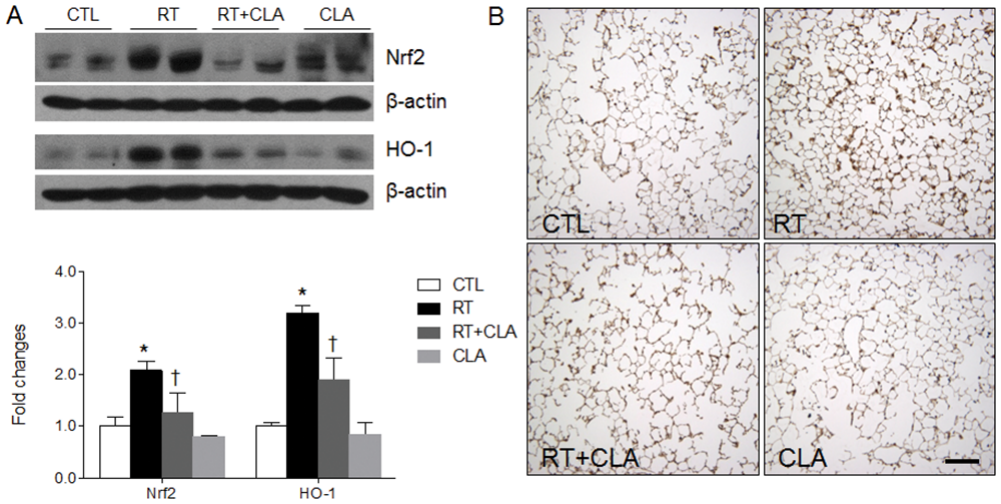
Effects of clarithromycin on Nrf2 and HO-1 expression levels and on HO-1 immunoreactivity in irradiated lungs of mice. (A) Nrf2 and HO-1 expression levels in lungs of control (CTL), radiation only (RT), radiation + clarithromycin (RT+CLA), and clarithromycin only (CLA) animal groups. Densitometry values were normalized to β-actin and data are presented as mean ± SEM (n = 2–6 mice per group). *p<0.05 vs CTL mice; †p<0.05 vs RT mice. (B) Immunostained HO-1 in lung tissue by group. Scale bar = 100 μm.

To evaluate the defense pathway of Nrf/HO-1 in irradiated lungs and its inhibition by CLA, western blot analyses using antibodies to Nrf2 and HO-1 were performed. The expression levels of Nrf2 and HO-1 in lungs of RT mice (vs CTL mice) were elevated. Significant reductions in Nrf2 and HO-1 expression levels were achieved with CLA administration (Fig 4A), and immunohistochemistry confirmed that increased post-irradiation immunoreactivity of HO-1 was inhibited by CLA (Fig 4B). As shown in Fig 4A, histological analysis showed that the percentage of mean intensity of HO-1 in RT mice was 362.61 ± 17.31. However, the percentage in RT+CLA mice (261.26 ± 10.20) was significantly reduced compared to RT mice (*p* < 0.05).

### Impact of CLA administration on TGF-β1, CTGF, and type I collagen gene expressions in irradiated lungs

**Fig 5:**
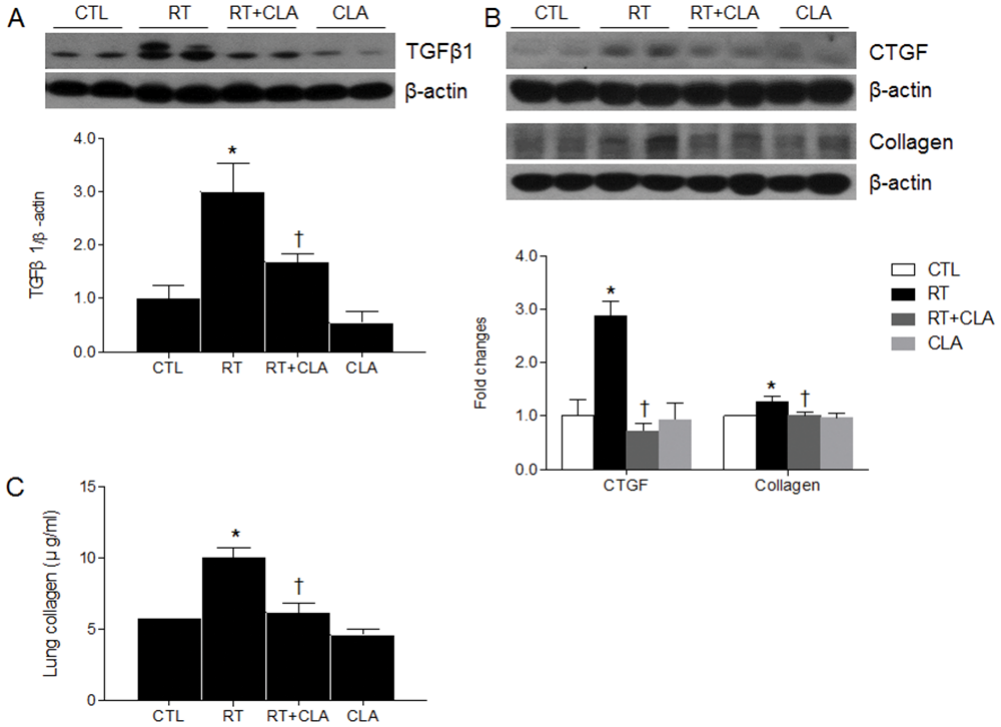
Effects of clarithromycin on TGFβ-1, CTGF, and collagen type I gene expressions and on tissue collagen concentration. (A) TGFβ-1 expression in lungs of control (CTL), radiation only (RT), radiation + clarithromycin (RT+CLA), and clarithromycin only (CLA) animal groups. (B) CTGF and collagen type I gene expressions in lungs of respective groups. Densitometry values were normalized to β-actin and data are presented as mean ± SEM (n = 2–6 mice per group). (C) Collagen concentration in lung tissue by group (n = 2–9 mice per group, Sircol collagen assay). *p<0.05 vs CTL mice; †*p*<0.05 vs RT mice.

To evaluate whether CLA could attenuate RILI-induced fibrosis, we assessed collagen synthesis-related proteins. We performed western blots using antibodies to TGF-β1, CTGF, and type I collagen. Consistently and significantly, expression levels of TGF-β1, CTGF, and type I collagen gene were increased in lungs of RT mice but were reduced in lungs of RT + CLA mice (Fig 5A and 5B). By Sircol collagen assay, the concentration of collagen in lungs of RT mice (vs CTL mice) was significantly higher but was reduced by CLA administration (Fig 5C).

## Discussion

The pathogenesis of radiation-induced tissue injury is complex and the exact mechanism was not fully understood [19]. The pulmonary fibrosis that follows thoracic irradiation is attributable to inflammation. Inflammation of injured lung tissues then necessitates a process of repair with repetitive injury-repair cycles contributing to subsequent pulmonary fibrosis. Generation of reactive oxygen species (ROS) is another significant factor in radiation-induced pulmonary fibrosis [20]. In this context, the observed effects of post-irradiation CLA administration in mice may be summarized as follows: 1) histologic manifestations of radiation injury to the lung (i.e., the influx of macrophages), alveolar epithelial changes, and fibrosis, were diminished; 2) radiation-related activation of the pro-inflammatory cytokine, TNF-α, and its receptors (TNFR1, TNFR2) was attenuated; 3) modulation of the NF-κB-mediated inflammatory pathway was evident; 4) modulation of Nrf2 signaling and reduced post-irradiation oxidative damage were also apparent; and 5) the TGF-β-mediated fibrosis pathway was inhibited, limiting subsequent collagen synthesis.

Alveolar macrophages produce a variety of pro- and anti-inflammatory cytokines, ROS, and chemokines and are pivotal in regulating pulmonary inflammation and repair [21]. The apoptosis of macrophages contribute to radiation-induced inflammation and fibrosis [22]. In the current study, microscopic findings and scores demonstrated that radiation-induced influx of alveolar macrophages was effectively reduced by CLA administration. Likewise, collagen synthesis in irradiated lung tissue and grades of pulmonary fibrosis were attenuated by the use of CLA. In addition, radiation-induced active caspase-3 expression was reduced by CLA administration. These findings indicate that CLA may alleviate radiation-induced pulmonary fibrosis through impaired macrophage activity-mediated inflammatory and repair processes, similar to observations of Nakanishi et al. These investigators found that CLA administration inhibited influx of alveolar macrophages in mice as a consequence of smoking [23].

TNF-α is a key cytokine in RILI, possessing both pro-inflammatory and immunomodulatory properties [24]. The interplay between TNF-α and TNFR is also crucial in regulating inflammatory and immune responses [25]. Previous studies indicate that TNF-α blockade inhibits bleomycin-induced pulmonary fibrosis in an animal model [26] and that TNF-α receptor knockout mice are protected from asbestos-induced pulmonary fibrosis [27].

In this study, overexpression of TNF-α, TNFR1, and TNFR2 in the lungs of mice after thoracic irradiation was curtailed by CLA administration, underscoring the efficacy of this drug in minimizing radiation-induced pulmonary inflammation. According to Bertok et al., the role of TNFR1 is critical in TNF-mediated responses of pulmonary endothelial cells of mice, with TNFR2 assuming lesser importance [28]. On the other hand, former colleagues of ours have shown that TNFR2 expression in lungs of rats is not affected by radiation [29]. Timing of post-irradiation determinations may have some bearing, given that our animals were sacrificed at week 16, as opposed to week 8 in another study. Although an explanation for the radiation-induced overexpression of TNFR2 observed here cannot be offered, the present study results in inhibition of both TNFR1 and TNFR2 expression levels when CLA is administered.

In the current study, radiation also triggered overexpression of acetylated NF-κB and its target proteins (COX-2, VCAM-1, and MMP-9) whereas expression was inhibited by CLA administration. NF-κB is an important transcription factor that regulates inflammatory responses and innate immunity [30], and in airway epithelial cells, its role in inflammation and injury is critical [31]. Previous studies have demonstrated that CLA administration inhibits the NF-κB pathway *in vitro* [32,33]. As such, inhibition of NF-κB-mediated cytokines (i.e., COX-2, VCAM1, and MMP9) may be a decisive means of preventing RILI.

Nrf2 is a transcription factor that induces antioxidant and detoxifying proteins critical in cytoprotection from oxidative injury [34]. Cho et al. discovered that Nrf2-deficient mice showed reduced pulmonary expression of various antioxidant enzymes, rendering them susceptible to hyperoxia-induced lung injury [35] and increasing their susceptibility to the pulmonary inflammation and fibrosis induced by bleomycin [36]. Recent work by Kim et al. further revealed that augmenting Nrf2 signals mitigated radiation-induced myelosuppression and mortality in mice [37]. Meanwhile, HO-1 is a microsomal enzyme that catalyzes the degradation of heme and presumably protects against oxidative damage. In one particular study, increased HO-1 levels helped to protect against hyperoxic lung injury in rats [38]. Because oxidative stress is central in a variety of fibrotic diseases, including idiopathic pulmonary fibrosis, cardiac fibrosis, and systemic sclerosis [39], our findings suggest that CLA may have a major impact on RILI prevention by reducing oxidative damage.

TGF-β is a representative cytokine that mediates fibrotic diseases (including radiation-induced pulmonary fibrosis) and is produced by a variety of cells (e.g., inflammatory, epithelial, and endothelial). Activated TGF-β contributes to production of collagen, other matrix molecules, and growth factors by activating myofibroblasts [40]. The significance of TGF-β in radiation-induced pulmonary fibrosis was demonstrated by Anscher et al. who found that administration of anti-TGF-β antibody attenuated RILI in the lungs of rats [41]. In this study, the anti-fibrotic effects of post-irradiation CLA administration were confirmed by various means: histologic fibrosis scores; expression levels of TGF-β1 CTGF, and type I collagen synthesis; and quantification of lung tissue collagen.

Effects of CLA on the treatment of some pulmonary diseases such as diffuse panbronchiolitis and cystic-fibrosis were well established. Immunomodulatory effects rather than anti-bacterial effects are important action mechanism of macrolide and thus, long-term duration of treatment is usually needed [13]. CLA suppresses productions of CXC-chemokines and various cytokines including interleukin-4, 5, 8, and 13 [42]. Furthermore, CLA decreased neutrophil influx and accumulation of other inflammatory cells [14,43]. Balancing immunological responses via CLA administration may be attributable to the preventive effects of CLA on RILI in this study. Further studies regarding immunomodulatory effects of CLA in RILI model are needed.

Several limitations of this study are acknowledged. (i) A single thoracic irradiation rather than fractionated irradiation was used in this study. Fractionated thoracic radiotherapy is standard method of radiation for the treatment of lung cancer. Some animal studies used fractionated radiation regimen that is clinically relevant method compared with single radiation [44–46]. It will be better to use fractionated radiation method in animal experiments. However, fractionated radiation is practically more difficult to perform than single radiation, and so many studies used a single thoracic radiation method in animal researches [47–50]. (ii) Serial changes in histopathologic features and cytokine expression levels following the single radiation exposure were not monitored. Some researchers have separately evaluated RILI in acute pneumonitis and chronic fibrosis periods (despite an uncertain transition point). Documentation of events over time may help to elucidate the precise mechanism of CLA in preventing RILI. (iii) There is also a chance that the dose of radiation (18-Gy) was excessive. Optimal dosing was not established for this animal model, and in similar studies of irradiated mice, lung exposures have varied (6-25-Gy) [49–52]. Experiments conducted at lower radiation doses are needed. (iv) Dose-dependent effects of CLA were not assessed. Although we are unaware of any study addressing this issue in RILI, Nakanishi et al. did indicate that the efficacy of CLA in preventing smoking-related emphysema was dose-dependent [23]. Optimal dosing of CLA to prevent RILI in both animals and human therefore awaits further study. Of note, the dose of CLA our test mice received (100 mg/kg) carried no significant side effects, relative to CTL group status. (v) Because this is the first known animal study evaluating the effects of a macrolide in RILI prevention, we are also mindful that cause-and-effect relationships among various markers of inflammation, fibrosis, and oxidative injury are still unclear.

## Conclusion

The current study confirms that CLA effectively reduces the severity of RILI in mice by mitigating pulmonary inflammation, fibrosis, and oxidative damage. Given these effects in our animal model, it is feasible that CLA may confer radioprotection in humans as well.

## Supporting Information

S1 ARRIVE Checklist ARRIVE Checklist. (PDF)
